# Lyophilized royal jelly preparation in nanoscale and evaluation of its physicochemical properties and bactericidal activity

**DOI:** 10.1002/fsn3.3330

**Published:** 2023-03-22

**Authors:** Reza Ghadimi‐Garjan, Afshin Javadi, Hoda Jafarizadeh‐Malmiri, Navideh Anarjan, Hamid Mirzaei

**Affiliations:** ^1^ Department of Food Hygiene, Faculty of Veterinary, Tabriz Medical Science Islamic Azad University Tabriz Iran; ^2^ Faculty of Chemical Engineering Sahand University of Technology Tabriz East Azarbaijan Iran; ^3^ Food and Drug Safety Research Tabriz University of Medical Sciences Tabriz Iran

**Keywords:** 10‐hydroxy‐2‐decanoic acid, antibacterial activity, freeze‐drying, physicochemical properties, royal jelly

## Abstract

Royal jelly, due to its unique bioactive components, has special biological activities, but a great extent of its nutritional value is lost during processing and storage. Lyophilization, an effective preservation technique, can feasibly preserve the main bioactive compounds present in royal jelly. In this study, fresh royal jelly was subjected to the freeze‐drying process at a pressure and temperature of 100 Pa and − 70°C, respectively, for 40 h. The results obtained indicated that the pH, turbidity, total phenol content, and antioxidant activity of the royal jelly powder (RJP), during 3 months of storage at ambient temperature (30°C), were constant with values of 4.30, 1.634 (%A.U.), 0.617 (g/L), and 28.7 (%), respectively. Moisture content of the prepared RJP was less than 1%, while that of the fresh royal jelly was 70%. Furthermore, for the fresh royal jelly, the mentioned parameters were significantly (*p* < .05) decreased after 2 months of storage at freezer temperature (−20°C). GC–MS analysis indicated that the amount of 10‐hydroxy‐2‐decanoic acid (10H2DA) in RJP was 3.85 times more than that of fresh royal jelly. The obtained results also indicated that prepared RJP had a high bactericidal effect toward *Escherichia coli* and *Staphylococcus aureus*, with clear zone diameters of 12 and 15 mm, respectively. The present study provides a foundation for research on the potential application of prepared RJP and the development of dietary supplements and functional foods.

## Graphical abstract

Fresh royal jelly was subjected to the freeze‐drying process with a pressure and temperature of 100 Pa and − 70°C, for 40 h. Statistical analysis of the moisture content, turbidity, total phenol content, and antioxidant activity of fresh royal jelly and royal jelly powder (RJP) indicated that during 3 months of storage, these parameters were constant for the prepared RJP; however, for the fresh royal jelly, these parameters significantly (*p* < .05) decreased after 2 months of storage.

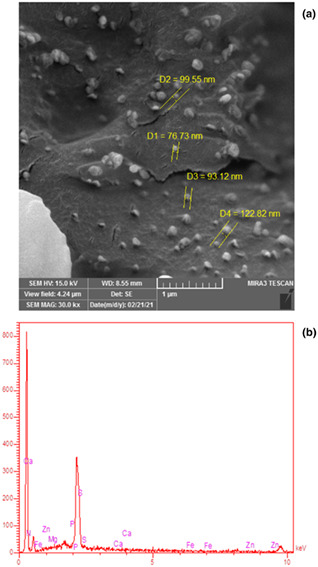

## 1 INTRODUCTION

Royal jelly is secreted by the hypopharyngeal glands of worker bees (Xue et al., 2017). Due to its unique bioactive components that include proteins, lipids, carbohydrates, amino acids, mineral salts, vitamins, enzymes, trace elements, and natural antibiotics, it has distinctive biological and pharmacological activities such as antimicrobial, antioxidant, and anticancer activities and blood pressure regulatory, anti‐inflammatory, immunomodulatory, anti‐allergic, and antiaging properties (Pavel et al., 2011). These unique characteristics of royal jelly in addition to its nutrient value can be drastically lost during processing and storage (Messia et al., 2005). Therefore, preservation of the nutritive value during preparation and production of the valuable products containing the royal jelly is an important issue for researchers and scientists. Royal jelly can be stored at the refrigeration temperature for 18 months, and its shelf life can be increased to 24 months at a storage temperature of −17°C. Royal jelly in its lyophilized and powder form can be easily stored at cold or room temperatures for long time (Messia et al., 2005).

In fact, royal jelly contains 60%–70% water, 12%–15% crude protein, 10%–16% total sugar, 3%–6% lipids, vitamins, salts, and free amino acids: more than 80% of the proteins in royal jelly are major royal jelly proteins (MRJPs), especially MRJP1‐9 (Xin et al., 2016). The Reduction of water content and storage temperature of the royal jelly decreases the biological and biochemical reactions between its main bioactive compounds, and most of the biological and physicochemical activities of the royal jelly remains constant. (Guo et al., 2021). Several studies have been done to evaluate different preservation methods with minimal loss in nutrition value and properties for royal jelly, such as storage of royal jelly mixed with honey at the refrigeration temperatures, and lyophilization of the royal jelly (Dzung, 2014). Lyophilization is a preservative process in which the product very quickly freezed and then subjected to a vacuum, which removes the major water content through sublimation (Messia et al., 2005).

In royal jelly, 10‐hydroxy 2‐decanoic acid (10H2DA) is the most important organic fatty acid that is known as an indicator for its quality, and most of the royal jelly biological activities are related to this fatty acid (Choi & Lee, 2013). Several studies indicated that the amount of 10H2DA in the lyophilized royal jelly is three times higher than that in fresh ones (Popescu et al., 2008; Sabatini et al., 2009). Because of the sensitivity of fresh royal jelly to environmental stresses such as light and temperature, lyophilization can effectively preserve main bioactive compounds present in it, such as volatile compounds, which at higher temperatures can easily evaporation (Dzung & Van Suc, 2015). Numerous studied have shown that lyophilized royal jelly has a shelf life of 18 months at room temperature, without significant changes in the amount of its main bioactive compounds and its biological activities (Choi & Lee, 2013; Messia et al., 2005).

Therefore, the main objectives of this study were to (i) produce royal jelly powder using the freeze‐drying technique, (ii) investigate of the physicochemical properties of fresh and lyophilized royal jelly powder during storage, and (iii) assess the antibacterial activity of lyophilized royal jelly against different bacteria strains.

## 2 MATERIALS AND METHODS

### 2.1 Materials

Fresh royal jelly was purchased from the local market in Tabriz (Iran) and stored at −20°C until further analyses and processes. 2, 2 diphenyl 1‐picrylhydrazyl and all other chemicals and reagents used for the study were of analytical grade and purchased from Merck. Plate count agar was also provided from Merck. Two bacterial strains, namely *Staphylococcus aureus* (PTCC 1764) and *Escherichia coli* (PTCC 1330), were obtained from the Persian type culture collection.

### 2.2 Royal jelly powder (RJP) preparation

In order to prepare RJP, the obtained fresh royal jelly was placed in a laboratory freezer that was adjusted at −20°C for 24 h and transferred to a freeze‐dryer (Zist Tajhiz Azma Co.) with chamber conditions of pressure and temperature of 100 Pa and −70°C, respectively, for 40 h. The formed RJP was then stored in a closed bottle at ambient temperature (30°C) for further analyses.

### 2.3 Analyses

To compare the quality of the prepared RJP with that of the fresh royal jelly, the fresh royal jelly was stored at freezer temperature (−20°C) and the RJP at room temperature (27°C) for 3 months. For preparation of dissolution samples, 1 g of each fresh and lyophilized royal jelly powder was added into 20 mL of distilled water and shaken.

#### 2.3.1 Nutritional values of the fresh royal jelly and RJP

Sugar, protein, lipid, moisture, and ash contents of the provided royal jelly and its prepared powders were assessed as the main nutritional values. In order to measure the protein content of the samples, the micro‐Kjeldahl technique was used. In this method, by measuring the total amounts of nitrogen in the samples, and using a coefficient of 6.25, total amounts of protein were obtained (Almeida‐Muradian et al., 2005). The lipid content of the samples was measured using a Soxhlet extraction apparatus (Pouya electric, 400‐PSU, Esfahan, Iran) with ether as the solvent at a boiling temperature of 60°C (Choudhury & Rahman, 1973). Total, reducing, and non‐reducing sugars in the samples were determined using Fehling's test, as describe by Aljohar et al. (2018).

The moisture content of the fresh royal jelly and prepared RJP were measured as follows: 1 g of the sample was placed in the oven (UF55Memmert) adjusted at 100 ± 5°C for 3 h, cooled in a desiccator, and then weighed. This procedure was repeated at 1 h drying intervals until the sample weight became constant (Nabas et al., 2014). The moisture content was calculated using the following equation:

(1) $$\text{Moisture content}\;\left(\%\right)=\left[\left(W_{i}-W_{f}\right)/W_{i}\right]\times 100$$

where *W*_i_ and *W*_f_ are the initial and final weights of the sample.

To determine the ash content of samples, 5 g of the pollen and its prepared powder were placed in ceramic crucible, weighed (*S*_i_), and put in a laboratory furnace adjusted at 600°C, to remove all moisture and volatiles (as vapor) and organics (as carbon dioxide and oxides of nitrogen). After 3 h, the final weight of the crucible containing the remaining inorganics (*S*_f_) was weight and the ash content was calculated using the following equation (Isik et al., 2019):

(2) $$\mathrm{Ash}\;\text{content}\;\left(\%\right)=\left[\left(S_{i}-S_{f}\right)/S_{i}\right]\times 100\;\left(1\right),$$

#### 2.3.2 Scanning electron microscopy and energy‐dispersive X‐Ray of the prepared RJP

The morphology of the prepared RJP was assessed by SEM (MIRA3 FEG‐SEM, TescanOrsay Holding, Thailand). Samples were mounted on a specimen stub, with double‐sided adhesive carbon tapes. The specimens were coated with gold and observed with a voltage acceleration of 30 kV at ×1,000,000 magnifications. The distribution of elements of royal jelly samples was detected using an energy‐dispersive X‐Ray (EDX) analysis. EDX element analysis can effectively indicate the existence of the components, and this is suitable to study the presence of the elements in the structure of the prepared RJP, according to the pattern of dissemination for these elements. In order to supply a preferable electrical conduction, the samples were plated with a thin covering of gold. EDX analysis for the prepared RJP was done using the method described by Sayyar & Jafarizadeh‐Malmiri (2019).

#### 2.3.3 pH and turbidity

pH and turbidity of the samples in the solution state were measured using a laboratory pH meter (pH lab 827‐Swiss) and an ultraviolet–visible spectrophotometer (250–800 nm, Perkn Elmer's Co.) adjusted at 625 nm, respectively (Ahdno & Jafarizadeh‐Malmiri, 2017).

#### 2.3.4 Antioxidant activity

The free radical scavenging method (DPPH) was used to measure the antioxidant activity. For this purpose, 0.004% DPPH solution in methanol was prepared and 2 mL of the sample added to that and mixed. Then, the prepared mixture solution was kept in the darkroom for 15 min. The control sample was the prepared DPPH solution without the sample. The measurements of UV–visible spectroscopy (250–800 nm, Rodgua 6705‐UK) were performed, and the absorbance of the samples was read at 517 nm. The percentage of free radical scavenging of DPPH was calculated using the following equation:

(3) $$I\%=\frac{A_{\text{Control}}-A_{\text{Sample}}}{A_{\text{Control}}}\times 100,$$

where *A*_Control_ is the absorption of the control sample, *A*_Sample_ is the absorption of the sample, and I% is the inhibition (Arjabi et al., 2021).

#### 2.3.5 Phenolic compounds

Phenolic compounds were measured using the spectrophotometric method with Folin–Ciocalteu reagent according to the method of Ghavidel et al. (2021). In this analysis, standard solutions of gallic acid with different concentrations (50, 100, 150, 200, 250, and 300 mg/g) in ethanol (70% V/V) were prepared, and 0.5 mL of these solutions were mixed with 2.5 mL of Folin‐Ciocalteu reagent, and during mixing, 2 mL of sodium carbonate (7.5% W/V) was added to the solutions. The samples were then placed in a dark room for 30 min, and the absorbance was measured using the UV–Vis spectrophotometer adjusted at 760 nm and the standard curve was plotted. To determine the total phenol content of fresh royal jelly and RJP, 0.5 mL of the samples, in solution form, was added into the prepared mixture solution containing 2 mL of sodium carbonate and 2.5 mL of Folin‐Ciocalteu reagent, and after 30 min, the absorbance of the solutions was measured at a wavelength of 760 nm. The results were expressed as g per liter of gallic acid.

#### 2.3.6 Infrared spectrometry (FTIR)

To identify the main functional groups in fresh and lyophilized royal jelly, Fourier‐transform infrared (FTIR) spectroscopy was performed. The FTIR spectra of the samples was provided by FT‐IR instrument (S8400 instrument) with potassium bromide (KBr) and a resolution of 4/cm, with the wavenumber range of 400–4000/cm (Ezazi et al., 2021).

#### 2.3.7 Gas chromatography (GC–MS)

The main bioactive compounds of the fresh and lyophilized royal jelly were detected utilizing a GC–MS (Agilent 6890). The GC was connected to a mass spectrometer (5973, Agilent) and the existed 10‐hydroxy 2‐decanoic acid (10H2DA) in the samples was qualitatively detected. The capillary column (HP‐88 MS) with a length of 100 meters, diameter of 0.25 mm, and layer thickness of 0.5 mm was utilized. The temperature of the injection and oven parts were 250 and 100°C at the beginning of the assay, respectively. Helium gas (99%) was used as a carrier gas with a velocity of 1 mL/min. The detector voltage was 1700 V (Saemi et al., 2021).

#### 2.3.8 Antibacterial activity assay

Bactericidal activity of the samples was assessed via the agar well diffusion method. The selected bacteria strains were cultured in PCA medium incubated at 37°C for 24 h. A 0.5 McFarland standard was used to adjust amount of bacteria in the suspensions to 10^8^ bacteria in 1 mL. After that 100 μL of the prepared bacterial suspension was poured and spread on the surface of the provided and set culture medium in the plates. Several holes with a diameter of 5 mm were created in the agar surface, and 20 μL of the samples were placed into the holes. The zones of growth inhibition around the holes were measured after 24 h of incubation at 37°C and diameter of the formed clear zone was noted as bactericidal activity of the samples. Chloramphenicol was used as control (Ristić et al., 2000).

### 2.4 Statistical analysis

All experiments were done in triplicates. The results of physicochemical and antimicrobial properties of the fresh royal jelly and the prepared PJP were assessed with Duncan multiple range tests (*α* = 0.05) to determine the difference among means with the confidence level of 95%.

## 3 RESULTS AND DISCUSSION

### 3.1 Appearance and nutritional values of the fresh royal jelly and RJP

The prepared RJP had a pale yellowish color. Furthermore, the results indicated that the prepared lyophilized RJP had a moisture content of less than 1%. Whereas, the provided fresh royal jelly had a water content of 70%. The results indicated that freeze‐dryer could efficiently remove more than 95% of water from fresh royal jelly. Nascimento et al. (2015) also reported that the water content of fresh royal jelly, which was 50%–60%, decreased to less than 1% using a freeze‐dryer. The results also indicated that fresh royal jelly had lipid, protein, total sugar and ash contents of 4%, 14%, 11%, and 1% (W/W), respectively. Furthermore, fructose, glucose, and sucrose contents of fresh royal jelly were 4.5%, 3.8%, and 2.7% (W/W), respectively. However, prepared RJP had lipid, protein, total sugar and ash contents of 9%, 32%, 26%, and 3% (W/W), respectively. The results also indicated that fructose, glucose, and sucrose contents of the prepared RJP were 13.2%, 11.8%, and 10.6% (W/W), respectively.

### 3.2 pH and turbidity of the fresh and lyophilized royal jelly

The pH of fresh royal jelly stored in the freezer for 3 months and the pH of lyophilized royal jelly stored at room temperature for 3 months are provided in Table 1. As shown in Table 1, during 3 months of storage, the pH of lyophilized RJP stored at room temperature was constant, while, in the case of fresh royal jelly, the pH was significantly (*p* < .05) decreased after 2 months of storage in the freezer. The acidic pH of the fresh and prepared powder of royal jelly can be related to the presence of acidic compounds in royal jelly, such as 10H2DA, benzoic acid, and other carboxylic acid compounds. Furthermore, the bactericidal activity of royal jelly is related to its acidic pH (Popescu et al., 2008).

**TABLE 1:** pH and turbidity of fresh royal jelly and the prepared RJP stored in the freezer and room temperatures for 3 months, respectively.

Physicochemical properties	Fresh royal jelly			Lyophilized RJP		
	First month	Second month	Third month	First month	Second month	Third month
pH	4.22 ± 0.02^Ab^	4.20 ± 0.01^Ab^	4.16 ± 0.02^Bb^	4.30 ± 0.00^Aa^	4.30 ± 0.01^Aa^	4.30 ± 0.01^Aa^
Turbidity (A.U. %)	0.590 ± 0.022^Aa^	0.560 ± 0.014^Aa^	0.580 ± 0.013^Aa^	1.636 ± 0.012^Aa^	1.632 ± 0.008^Aa^	1.634 ± 0.002^Aa^

As clearly observed in Table 1, the turbidity of fresh royal jelly and its prepared powder using freeze‐dryer was constant during 3 months of storage. However, the turbidity of prepared RJP was higher than that of the fresh royal jelly, around three‐fold. This could be related to the lowest amount of water and the highest solid content of prepared RJP, as compared to those of the fresh royal jelly. The highest solid content of the RJP indicates that the concentration of the bioactive compounds in the RJP was higher than those of the fresh royal jelly, which that causes a significant difference between the physical, chemical, and biological properties of lyophilized RJP, compared to fresh royal jelly (Babaei et al., 2016).

### 3.3 Total phenolic content and antioxidant activity of the fresh and lyophilized royal jelly

Total phenol contents (g/L) of the fresh royal jelly stored in the freezer and lyophilized RJP stored at room temperature during 3 months are provided in Table 2. As clearly observed in this table, the total phenol content of prepared RJP was 3.52 times higher than that of fresh royal jelly, due to the same reason as mentioned for the turbidity (given in the section before). Furthermore, obtained statistical results indicated that during 3 months of storage at room temperature, the total phenol content of the RJP was constant; however, this value for the fresh royal jelly was decreased after 2 months of the storage at freezer temperature.

**TABLE 2:** The chemical properties of fresh royal jelly stored in the freezer and lyophilized royal jelly stored at room temperature for 3 months.

Chemical properties	Fresh royal jelly			Lyophilized RJP		
	First month	Second month	Third month	First month	Second month	Third month
Total phenol (g/L)	0.176 ± 0.003^Ab^	0.171 ± 0.002^Ab^	0.167 ± 0.001^Bb^	0.620 ± 0.001^Aa^	0.617 ± 0.005^Aa^	0.617 ± 0.003^Aa^
Antioxidant activity (%)	30.6 ± 0.13^Ab^	29.2 ± 0.11^ABb^	28.7 ± 0.21^Bb^	60.6 ± 0.39^Aa^	60.2 ± 0.12^Aa^	60.1 ± 0.27^Aa^

As can be seen in Table 2, the antioxidant activity of RJP was around 2 times higher than that of fresh royal jelly. The statistical analysis showed no significant difference between the antioxidant activity of prepared RJP during 3 months of storage at room temperature. Whereas, there was significant difference between the antioxidant activity of the fresh royal jelly stored at freezer temperature in the first and third months. There was a good correlation between the antioxidant activity and the total phenol content of the samples: the higher the total phenol contents, the higher the antioxidant activity. In fact, most of the antioxidant activity of the components was related to the amount of the phenolic compounds present (Ghavidel et al., 2021).

### 3.4 FTIR analyses of royal jelly samples

**FIGURE 1:**
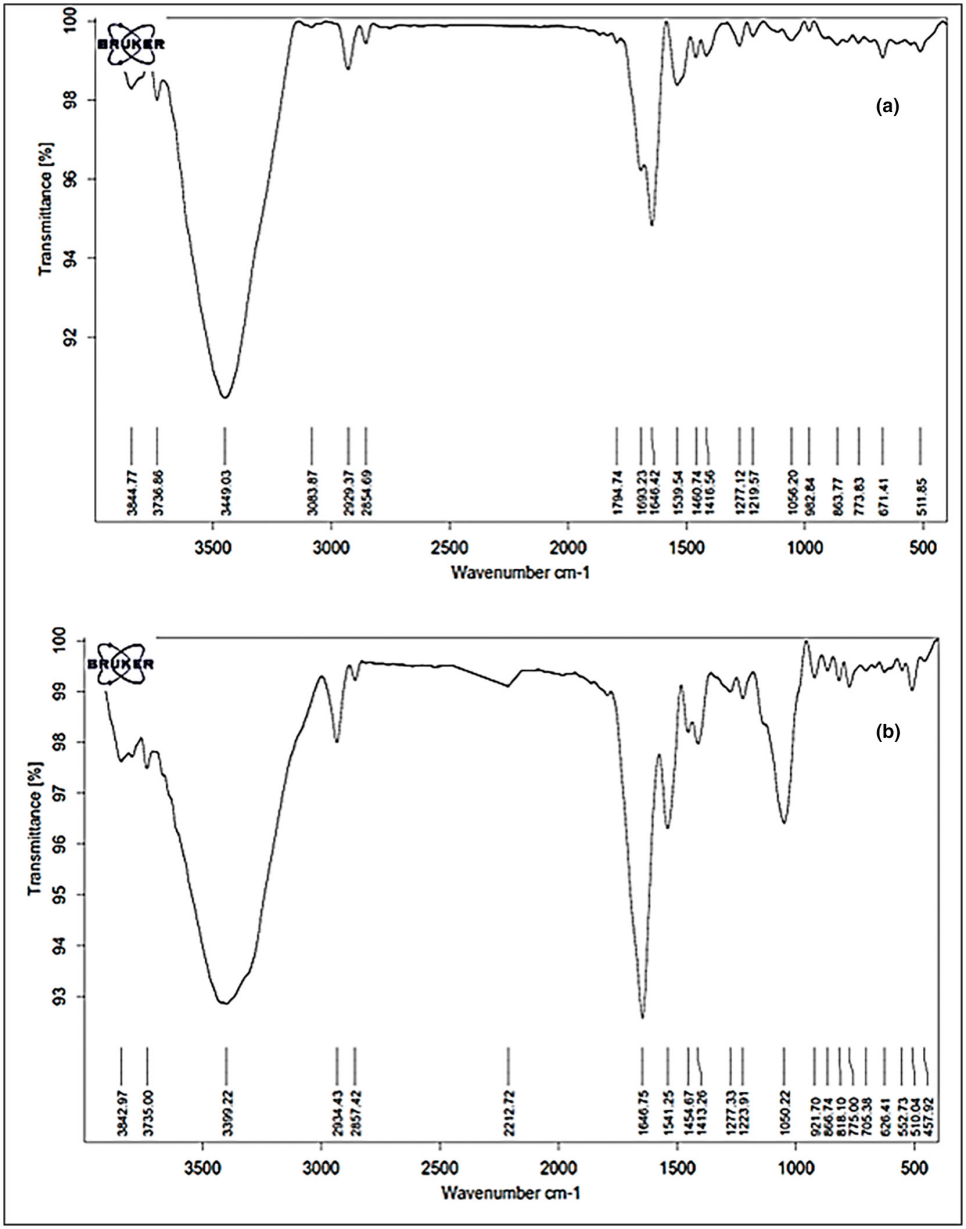
FTIR spectra of fresh royal jelly (a) and lyophilized RJP (b) at the beginning of storage.

Main functional groups in the fresh royal jelly and its lyophilized powder are shown in Figure 1a,b, respectively. As can be seen in Figure 1a, there are three main peaks centered at 3400, 2930, and 1640/cm in the FTIR spectrum of fresh royal jelly. The strong and broad absorption band at 3400/cm has been observed due to –OH stretching mode. The band at 2930/cm was assigned to the C‐C stretching of linkages of alkanes. The strong absorption bands at 1640/cm can be associated with the C=N stretching in amide groups. In addition to these highlighted peaks, there is an extra highlighted peak in the FTIR spectra of the prepared RJP, which was centered at 1050/cm (Figure 1b). This important peak was related to the RCO‐OH groups, due to the presence of carboxylic acids (Medina‐Torres et al., 2016). This peak could be strongly related to the presence of 10H2DA, benzoic acid, and gluconic acid in RJP, which were in high concentrations due to less moisture content and high solid content concentration in the RJP compared to the fresh royal jelly (Pavel et al., 2011). Furthermore, the amide groups in both samples were related to the proteins existing in fresh royal jelly and its powder. KDA‐57 protein, commonly known as royal actin, is the main protein of royal jelly and is the cause of the difference between worker bees and queens. This protein changes the bee larvae into queens (Sabatini et al., 2009). Tarantilis et al. (2012) studied the peak related to the amide functional group in the royal jelly. They evaluated the degradation of the royal jelly based on its protein degradation. Those results indicated that the royal jelly protein was destroyed by reducing the intensity of the amide peak during storage, and royal jelly had a shelf life of 3 days, 7 weeks, and 21 weeks at room temperature, 4°C, and −20°C, respectively. The results of the present study showed that the FTIR spectra of RJP after 3 months of storage at room temperature was similar to that in Figure 1b. However, the intensity of amid peak in the FTIR spectra of the fresh royal jelly stored at freezer temperature decreased as compared to that for the first month of storage at freezer temperature (Figure 1a). These results indicate the partial loss of protein in the fresh royal jelly after 3 months of storage.

### 3.5 Main bioactive compounds of the fresh royal jelly and its prepared powder

**FIGURE 2:**
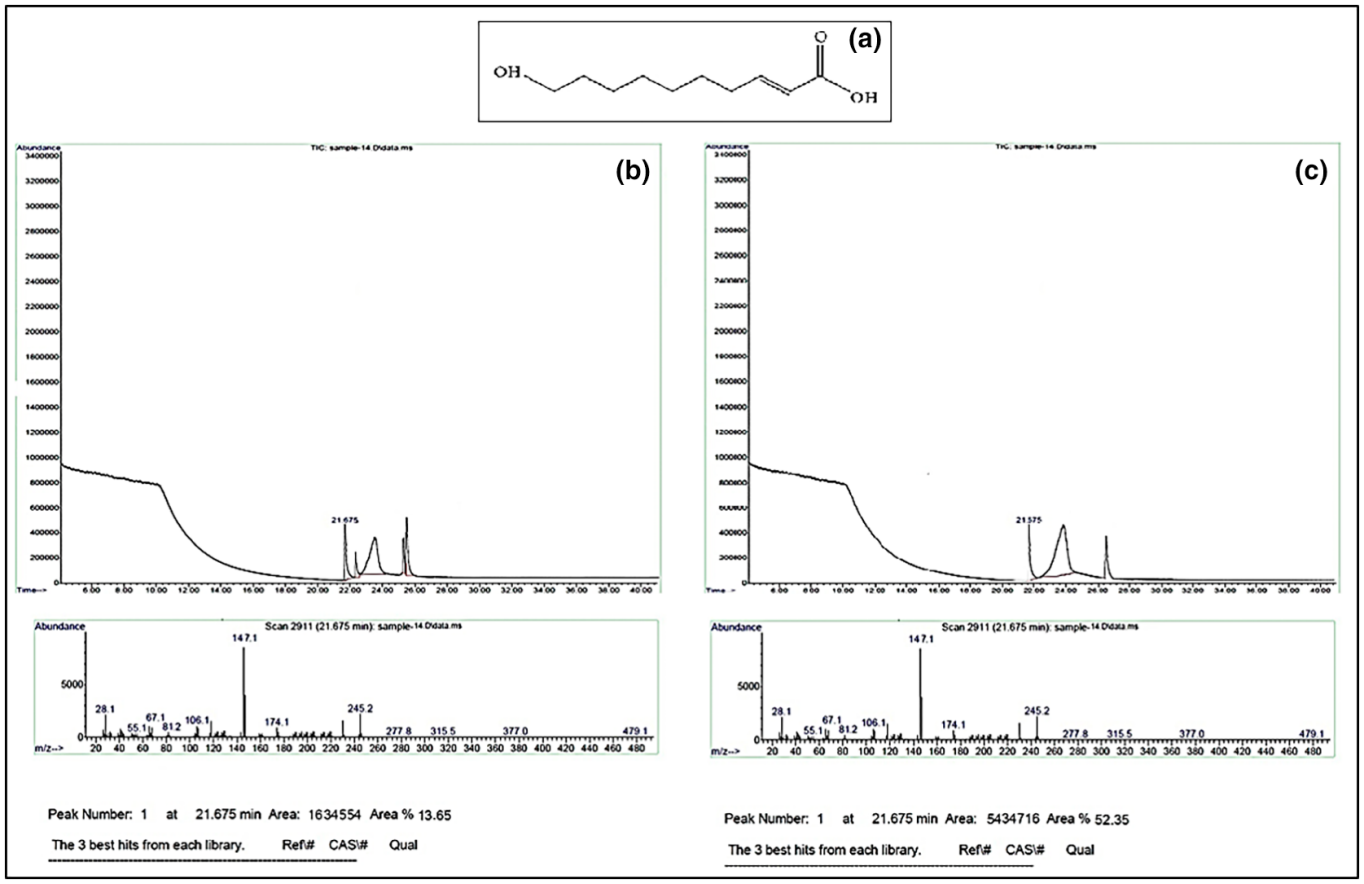
Chemical structure of 10H2DA (a), and the GC chromatograms of fresh royal jelly (b) and the prepared RJP (c) at the beginning of storage.

Figure 2a shows the chemical structure of 10H2DA, which is the most important bioactive of the royal jelly and is the main index of royal jelly quality. Based on GC chromatograms of fresh royal jelly (Figure 2b) and lyophilized RJP (Figure 2c), the main bioactive compounds are identified in royal jelly. As can be seen in both royal jelly samples, the resulting peak at 21.675 min belongs to the 10H2DA. Comparing the two GC chromatograms and the height of the peaks at the mentioned time (21.675 min), the concentration of 10H2DA in RJP was 3.85 times more than the concentration of 10H2DA in fresh royal jelly. After 3 months of storage, the amount of 10H2DA in lyophilized RJP was constant; however, its amount was decreased by 20% as compared to the beginning of the storage of fresh royal jelly in the freezer.

### 3.6 Antibacterial activity

**FIGURE 3:**
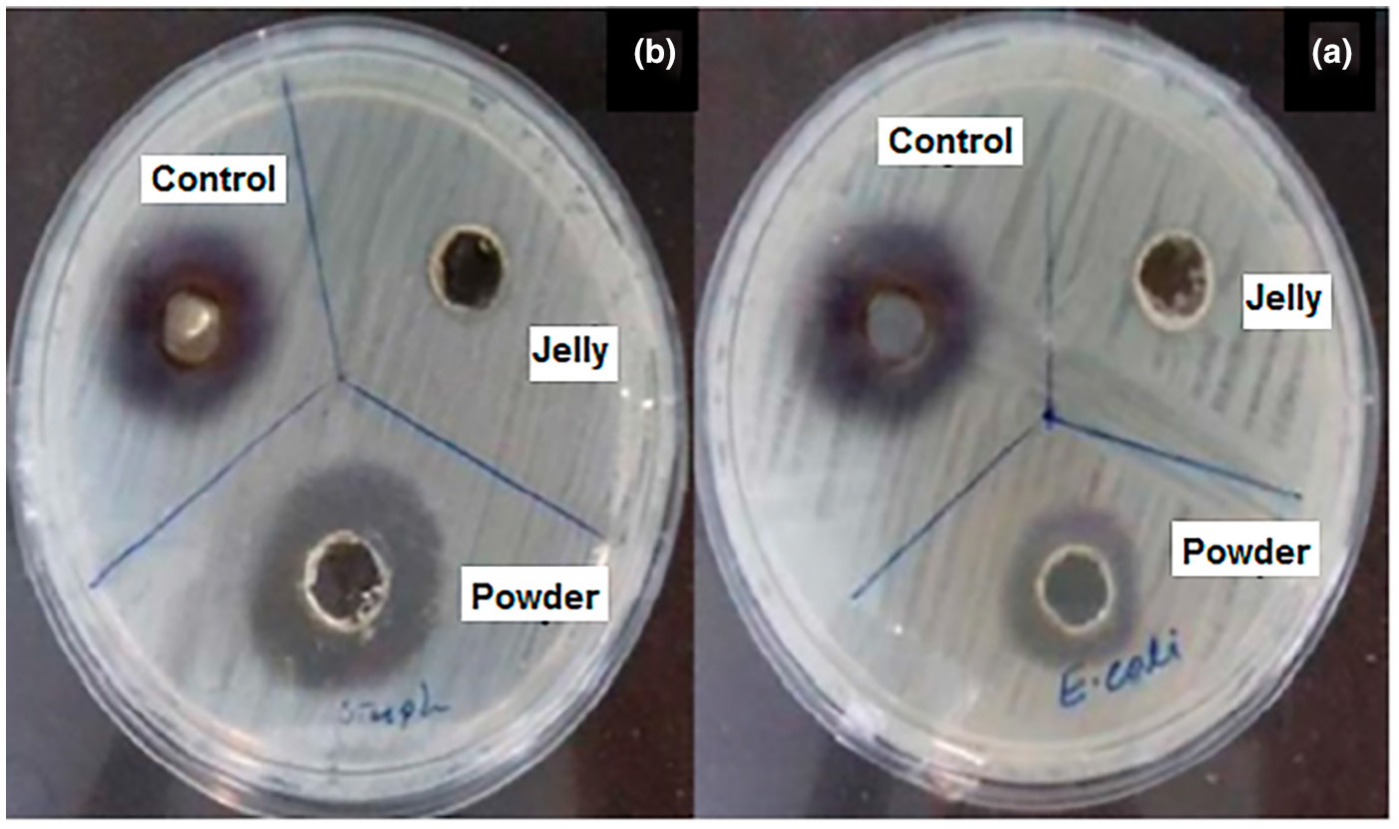
Antibacterial activity of fresh royal jelly, RJP, and control sample against *Escherichia coli* (a) and *Staphylococcus aureus* (b) after 3 months storage at different temperatures.

Figure 3a,b shows bactericidal activities of fresh royal jelly, its prepared powder, and control sample (chloramphenicol) toward two bacteria strains, namely *E. coli* and *S. aureus*, respectively. As clearly observed in this figure, fresh royal jelly had no antibacterial activity against both selected bacteria strains. On the other hand, the prepared RJP had high antibacterial activity against both gram‐positive and gram‐negative bacteria. Results indicated that the diameter of clear zones around the holes containing RJP in the plates amended with *E. coli* and *S. aureus* were 12 and 15 mm, respectively. Whereas, the diameters of clear zones for the antibiotic of chloramphenicol in the plates containing these two bacteria strains were same, 17 mm. The results demonstrated that the antibacterial activity of RJP against *S. aureus* was substantially higher than that of the lyophilized royal jelly against *E. coli*. Nascimento et al. (2015) found that the natural peptides, was the main factor in the antibiotic properties of royal jelly, and they also found that RJP had higher antibacterial activity compared to the fresh royal jelly. Furthermore, this can be attributed to the higher concentration of other chemical compounds such as royalisin, jeleines‐I, ‐II, ‐III, 10‐hydroxy 2‐decanoic acid (10H2DA), and other peptides. Another study also indicated that the primary mechanism of the antibacterial activity of the royal jelly may be due to the interaction of peptides with cell membranes (Fratini et al., 2016). Antibacterial peptides have a positive charge due to the presence of arginine, histidine, and lysine deposits, which allow them to react with anionic phospholipids of the cell membrane and cause cell destruction (Pavel et al., 2011). Obtained results also indicated that the antibacterial activity of RJP against gram‐positive bacteria was more than gram‐negative bacteria strain. On the other hand, gram‐negative bacteria were less sensitive to RJP. These results can be related to the difference between the cell wall structure of gram‐negative and gram‐positive species. The peptidoglycan layer in the gram‐negative bacteria cell wall is composed of lipoproteins, purines, and proteins, which can strongly protect the peptidoglycan layer against antibiotics (Akca et al., 2016; Azhdarzadeh & Hojjati, 2016).

### 3.7 Morphology and elemental profile of the prepared RJP

**FIGURE 4:**
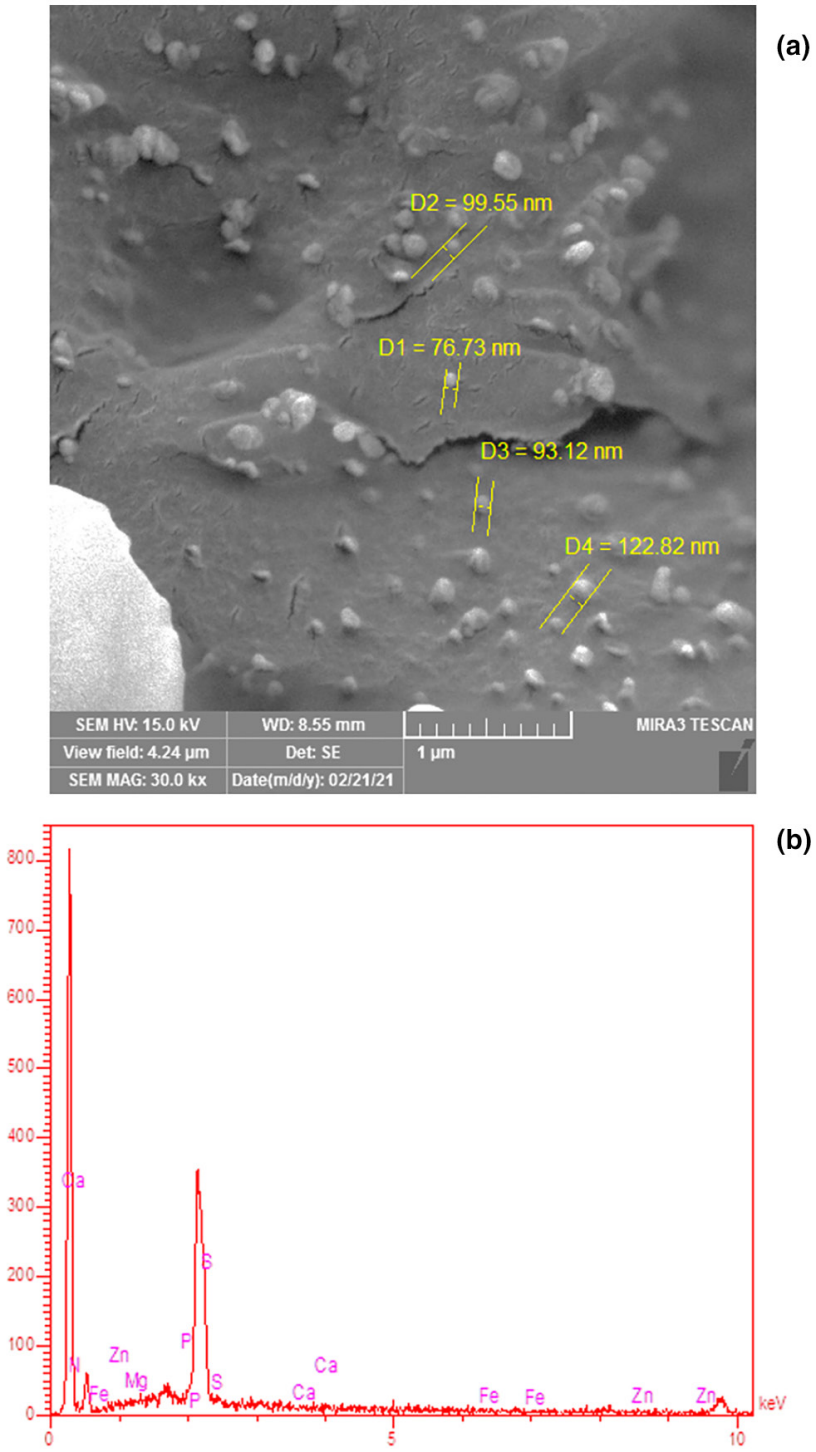
SEM image (a) and EDX elemental profile (b) of the lyophilized RJP after 3 months of storage at ambient temperature.

Shape and mean particle size of the prepared lyophilized RJP, SEM image, is shown in Figure 4a. As clearly observed in this figure, the spherical RJP with mean particle size ranging 70–150 nm was formed during freeze‐drying process. The spherical shape of RJP particles indicated that the particles had low surface energy with high stability. This could efficiently prevent undesirable phenomena such as particle agglomeration, as can be seen in the Figure 4a.

The results of the elemental analysis of lyophilized RJP is shown in Figure 4b. As can be seen in the figure, the nitrogen, magnesium, sulfur, phosphorus, zinc, iron, and calcium were the main elements existing in the prepared RJP. Among those, sulfur and calcium could be found in higher concentrations. It seems that the main reason for effectiveness of royal jelly on the skeletal tissue of the body, bones, and teeth is related to its high calcium concentration, and due to that, RJP has been widely used in traditional oriental medicine (Guo et al., 2021; Pavel et al., 2011). Royal jelly can stimulate osteogenesis by acting on estrogen receptors in living organisms (Kafadar et al., 2012). Furthermore, proline, lysine, aspartic acid, glutamic acid, serine, and β‐alanine are the major free amino acids in royal jelly, where nitrogen is the main element of those structure (Miyauchi‐Wakuda et al., 2019). Adenosine triphosphate is a major constituent of royal jelly, which acts as an energy carrier and energy transmitter in human cells. Liao et al. (2020) studied 45 types of the royal jelly and reported that the amount of adenosine in the different types of royal jelly varied from 5.9 to 2057.4 mg/kg. Phosphorus is an important component of royal jelly. Royal jelly contains a variety members of the vitamin B family and vitamins A, C, D, and E, among which thiamine (B1) is a prominent vitamin and sulfur is the main element of its chemical structure. Thiamine plays an important role in transmitting neural messages (Kamyab et al., 2020).

## 4 CONCLUSIONS

The results of the present study indicated that freeze‐drying was an effective way to prepare nano‐sized fine RJP, with the highest efficiency of 99%. This could effectively increase shelf life of the royal jelly in its powder form and in the room storage temperature, without any significant changes in its physicochemical, biological, and nutritional attributes. Furthermore, the results indicated that 10H2DA, the main valuable compound of the royal jelly that is more sensitive to the oxygen, temperature, and various environmental parameters, was preserved during the drying process and storage. The developed technique in this work provides numerous evidence supporting the utilization of RJP prepared using freeze‐dryer in the commercial production of functional beverage and foods. It also suggests that there is a need for optimization of the processing conditions to provide RJP with the highest antioxidant and antibacterial activities for further applications in cosmetics and pharmaceutics.

## AUTHOR CONTRIBUTIONS

Reza Ghadimi‐Garjan: methodology; Afshin Javadi: supervision; Hoda Jafarizadeh‐Malmiri: supervision, conceptualization, investigation, statistical analysis, writing original draft preparation; Navideh Anarjan; investigation, statistical analysis; and Hamid Mirzaei;rResources, investigation. All authors have read and agreed to the published version of the manuscript.

## FUNDING INFORMATION

This research has not received any sort of funding from any organization, in the public or private sectors.

## CONFLICT OF INTEREST STATEMENT

The authors declare no competing interests.

## Data Availability

The supporting datasets generated during and/or analyzed during the current study are available from the corresponding author on reasonable request.
